# National Preparedness Month — September 2017

**DOI:** 10.15585/mmwr.mm6636a1

**Published:** 2017-09-15

Every September, CDC, private and public health institutions, and approximately 3,000 government organizations support preparedness efforts and encourage Americans to take action before, during, and after an emergency. Every community in the United States should be ready to respond to an infectious disease outbreak, chemical or radiological release, or natural disaster (*1*). Public health systems should have the capacity to scale up and respond to the varying demands of public health emergencies (*2*).

Many emergencies happen without warning; it is important for all persons to take steps ahead of time to keep themselves and their loved ones safe and healthy. Research shows that only 46% of persons think a natural disaster is likely to occur in their community (*3*). It is vital to take immediate and appropriate actions in the event of an emergency.

This year, CDC’s Office of Public Health Preparedness and Response focuses on empowering individuals to better prepare for public health emergencies. The 2017 theme “The Power of Preparedness” highlights the importance of building and updating an emergency kit, having and reviewing an emergency plan, inspiring others to prepare, and taking immediate action to save lives. This issue of *MMWR* includes a report describing a series of unannounced mystery patient drills that were conducted in New York City emergency departments to assess response to potential infectious disease threats. Individual and community preparedness resources are available at https://www.cdc.gov/phpr/preparedness_month.htm.
